# Green radiology (part 1): environmental sustainability and energy consumption in medical imaging, with perspectives from Japan

**DOI:** 10.1007/s11604-026-02043-0

**Published:** 2026-07-10

**Authors:** Hideki Ota, Tsuneo Yamashiro, Kanako Kunishima Kumamaru, Soma Kumasaka, Aki Kido, Kumi Ozaki, Yumiko Kono, Minako Azuma, Tetsuya Fukuda, Noriko Aida

**Affiliations:** 1 Department of Diagnostic Radiology, Tohoku University Hospital, 1-1 Seiyo-machi, Aoba-ku, Sendai, 9808524 Japan; 2 Department of Radiology, St. Luke’s International Hospital, Tokyo, Japan; 3 Faculty of Health Data Science , Juntendo University, Chiba, Japan; 4 Department of Applied Medical Imaging, Gunma University Graduate School of Medicine, Maebashi, Japan; 5 Department of Radiology , Toyama University Hospital, Toyama, Japan; 6 Department of Radiology, Hamamatsu University School of Medicine, Hamamatsu, Japan; 7 Department of Radiology , Kansai Medical University, Hirakata, Japan; 8 Faculty of Medicine, Department of radiology, University of Miyazaki, Miyazaki, Japan; 9 Department of Radiology, National Cerebral and Cardiovascular Center, Suita, Japan; 10 Department of Diagnostic Radiology, Yokohama City University Graduate School of Medicine, Yokohama, Japan; 11 Department of Diagnostic Radiology, Shonan Kamakura General Hospital, Kamakura, Japan

**Keywords:** Green Radiology, Sustainable Radiology, Medical imaging, Environmental sustainability, Energy consumption, Life cycle assessment, Carbon footprint, Low-value imaging, Planetary health, Artificial intelligence

## Abstract

Climate change is an important public health challenge, and healthcare itself contributes to greenhouse gas emissions. Within healthcare, radiology is energy intensive because modern imaging services require high-power equipment, cooling systems, digital infrastructure, consumables, and continuous operational readiness. This review summarizes current evidence on the environmental footprint of radiology and discusses practical strategies for implementing Green Radiology, with particular attention to Japan. Life cycle assessment studies indicate that the operational phase of imaging systems, especially electricity use, is an important contributor to radiology-related emissions, while non-productive idle periods represent a key opportunity for mitigation. MRI, CT, interventional radiology, picture archiving and communication systems, workstations, and emerging artificial intelligence (AI)-related infrastructure each contribute to this footprint in different ways. Practical measures include equipment power management, workflow optimization, protocol optimization, energy-aware information technology management, careful implementation of AI, and reduction of low-value imaging, many of which may also improve operational efficiency and reduce costs. However, these strategies must be implemented with attention to emergency readiness, scanner warm-up requirements, diagnostic accuracy, patient safety, and clinical workflow. In Japan, high CT and MRI scanner density with relatively low per-scanner utilization may increase non-productive energy use, but broad equipment distribution also supports accessibility, regional equity, and emergency preparedness. At the system level, reducing the environmental footprint of radiology in Japan therefore requires balanced regional coordination, shared resource utilization, selective consolidation where appropriate, and effective allocation of limited radiologist resources. Green Radiology should aim to reduce avoidable environmental burden while maintaining diagnostic accuracy, patient safety, equitable access to medical imaging, and high-quality patient care.

## Introduction: planetary health and green radiology

**Fig. 1:**
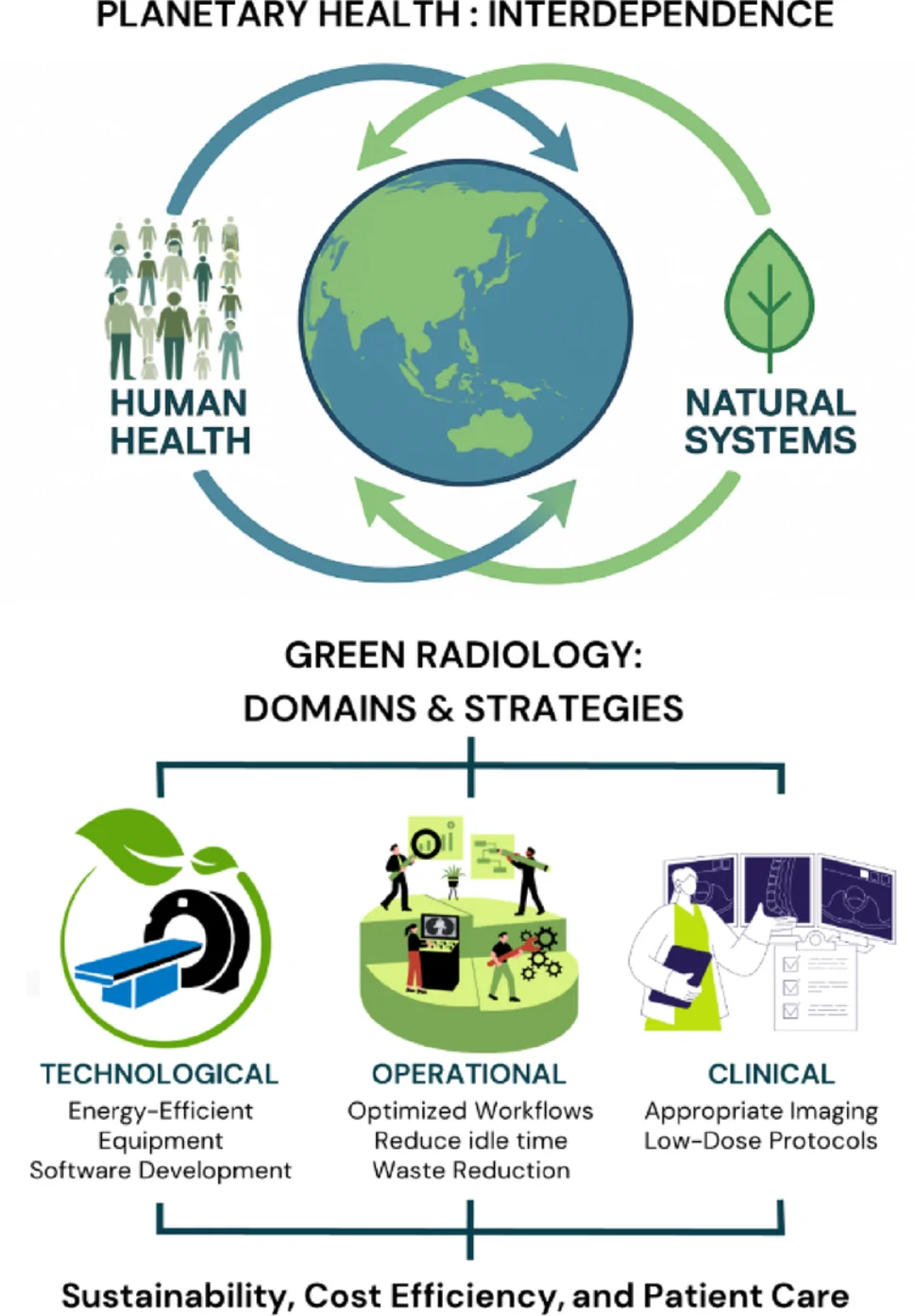
Planetary health considers the interdependence between human health and the Earth’s natural systems. Healthcare itself is also a contributor to environmental impact. Green radiology applies sustainability principles to medical imaging across technological, operational, and clinical domains to reduce environmental impact while maintaining clinical quality and economic efficiency

Planetary health recognizes the interdependence between human health and the health of the Earth’s natural systems [1–3] (Fig. 1). Climate change, driven largely by anthropogenic greenhouse gas (GHG) emissions, is widely regarded as a major global health threat [4]. It contributes to extreme weather events, worsening air quality, and shifts in disease patterns [2, 3, 5]. These impacts place increasing pressure on healthcare systems, making reduction of the environmental footprint of healthcare an important public health priority [3, 6]. Environmental footprint refers to the overall environmental burden associated with an activity, product, or system and encompasses multiple domains, including GHG emissions, energy consumption, resource depletion, water use, waste generation, and ecotoxicity-related impacts. Thus, reducing the environmental footprint of healthcare should be regarded not merely as an environmental initiative but as an essential public health responsibility aligned with the medical principle of *primum non nocere* (“first, do no harm”) [3, 7, 8].

Paradoxically, while the healthcare sector exists to protect and improve human health, it also contributes substantially to GHG emissions. In 2014, healthcare activities accounted for approximately 4.4% of total global GHG emissions [9, 10]. The carbon footprint, defined as the total amount of greenhouse gas emissions expressed as CO₂ equivalents across the life cycle of a given activity, system, or product, provides a useful framework for quantifying such environmental impacts. Among countries with the largest healthcare-related carbon footprints, the United States accounted for 27% of global emissions, followed by China (17%) and the European Union (12%), while Japan ranked fourth at approximately 5% [10]. Within Japan, the healthcare sector is estimated to contribute 4.6–7.6% of national GHG emissions [9–11].

Radiology services represent a particularly resource- and energy-intensive component of healthcare. The manufacturing, operation, and disposal of medical imaging equipment are estimated to account for approximately 0.8–1% of global carbon emissions [2, 12]. The energy consumption of cross-sectional imaging systems, particularly CT and MRI, can be substantial. For example, Heye et al. reported that the combined annual energy consumption of three CT scanners and four MRI systems in a single hospital exceeded 1.1 gigawatt-hours (GWh), roughly equivalent to the annual electricity demand of a town of approximately 850 people [13]. To put this into perspective at the individual scanner level, a CT scanner typically consumes energy comparable to that of about five four-person households annually, whereas MRI systems may correspond to approximately 26 households [13]. As demand for diagnostic imaging and image-guided interventions continues to increase worldwide, the environmental footprint of radiology is likely to grow unless effective mitigation strategies are implemented. In response to these challenges, Green Radiology, also referred to as Sustainable Radiology, has emerged as an important framework for integrating environmental sustainability into radiology practice. It encompasses environmental impacts of imaging technologies, operational processes, and clinical practices, and aims to minimize these impacts while maintaining diagnostic quality, patient safety, and equitable access to care (Fig. 1). Key objectives include improving energy efficiency, reducing carbon emissions and water-related impacts, optimizing resource use, minimizing waste, and promoting appropriate imaging practices in line with Choosing Wisely [14]. Beyond its environmental benefits, Green Radiology may also contribute to more efficient and cost-effective healthcare delivery by reducing waste and optimizing the use of energy and resources [15]. Green Radiology initiatives should be implemented in a manner that balances environmental objectives with high-quality patient care and healthcare system needs.

Recognizing the urgency of these challenges, major radiological societies have increasingly emphasized sustainability as a strategic priority [7, 16]. In Japan, this growing attention is reflected in the recent launch of the Sustainable Radiology Committee within the Japan Radiological Society and in editorials in the Japanese Journal of Radiology highlighting sustainability as an emerging priority for the radiological community [17]. Because environmental sustainability in medical imaging encompasses diverse issues, this review is presented in two parts. The present article (Part 1) focuses on conceptual frameworks for sustainability, life cycle assessment (LCA) of radiological services, modality-specific energy consumption patterns, and practical strategies to reduce energy use. It also discusses structural challenges relevant to radiological practice in Japan. A subsequent review (Part 2) will address environmental issues related to contrast media, including gadolinium- and iodine-based agents, plastic consumables used in contrast administration, and broader issues related to resource utilization.

## Frameworks for green radiology

Various frameworks have been proposed to support environmental sustainability in healthcare and radiology practice at different organizational levels. WHO frameworks provide overarching guidance for building climate-resilient health systems, as well as facility-level guidance for climate-resilient and environmentally sustainable health care facilities [18, 19]. Within clinical practice, sustainability can be implemented through quality-improvement methodologies such as Sustainable Quality Improvement (SusQI) [20], while radiology-specific frameworks such as EcoRad [21] provide practical approaches tailored to imaging services. These frameworks integrate environmental considerations with traditional goals of healthcare quality, cost efficiency, and patient care.

SusQI, developed by the Centre for Sustainable Healthcare in the United Kingdom, expands the conventional concept of healthcare value by incorporating environmental and social dimensions alongside clinical and economic outcomes [5, 20]. In radiology, applying the SusQI framework may create a “win–win–win” situation that benefits patients, healthcare systems, and the environment simultaneously [5].

A radiology-specific example is the EcoRad framework, proposed by Northrup et al., which introduces a “triple aim” for radiology departments: promoting environmental sustainability, reducing costs, and improving patient care [21]. The framework highlights that environmentally responsible practices—such as reducing energy use during non-clinical hours, adopting sustainable procurement practices, and avoiding low-value imaging—may also improve operational performance and financial efficiency in radiology departments.

Successful implementation of these frameworks requires organizational support and leadership. A systematic review by Federico et al. emphasized the importance of Green Transformational Leadership (GTL) among radiography managers and department chairs [22]. Leaders who incorporate environmental goals into strategic planning, promote staff education, and encourage sustainable practices can foster a departmental culture that supports environmentally responsible behavior among physicians, technologists, and nurses.

To support the practical implementation of these frameworks, certification systems have also been introduced to promote sustainable practices in radiology departments. One example is the Green Imaging Department (Green ID) certification developed by the European Society of Radiology (ESR) [23]. This initiative provides a structured framework for evaluating sustainability practices across multiple domains, including education and training, energy consumption, waste management, clinical decision support, procurement, and departmental governance. Radiology departments can obtain certification by demonstrating measurable actions in these areas, thereby promoting systematic implementation and monitoring of sustainable radiology practices. Such certification systems may help standardize sustainability practices and provide measurable benchmarks for radiology departments.

Although these frameworks provide valuable guidance for implementing sustainable radiology, the supporting evidence differs in nature and strength. Many recommendations are currently derived from policy guidance, conceptual models, quality-improvement initiatives, expert consensus, or observational experience rather than prospective comparative studies, and their effectiveness may vary depending on local organizational and healthcare system factors. Further research is needed to evaluate whether these frameworks can achieve measurable outcomes in sustainable radiology.

## Life cycle assessment and environmental impact in radiology

**Fig. 2:**
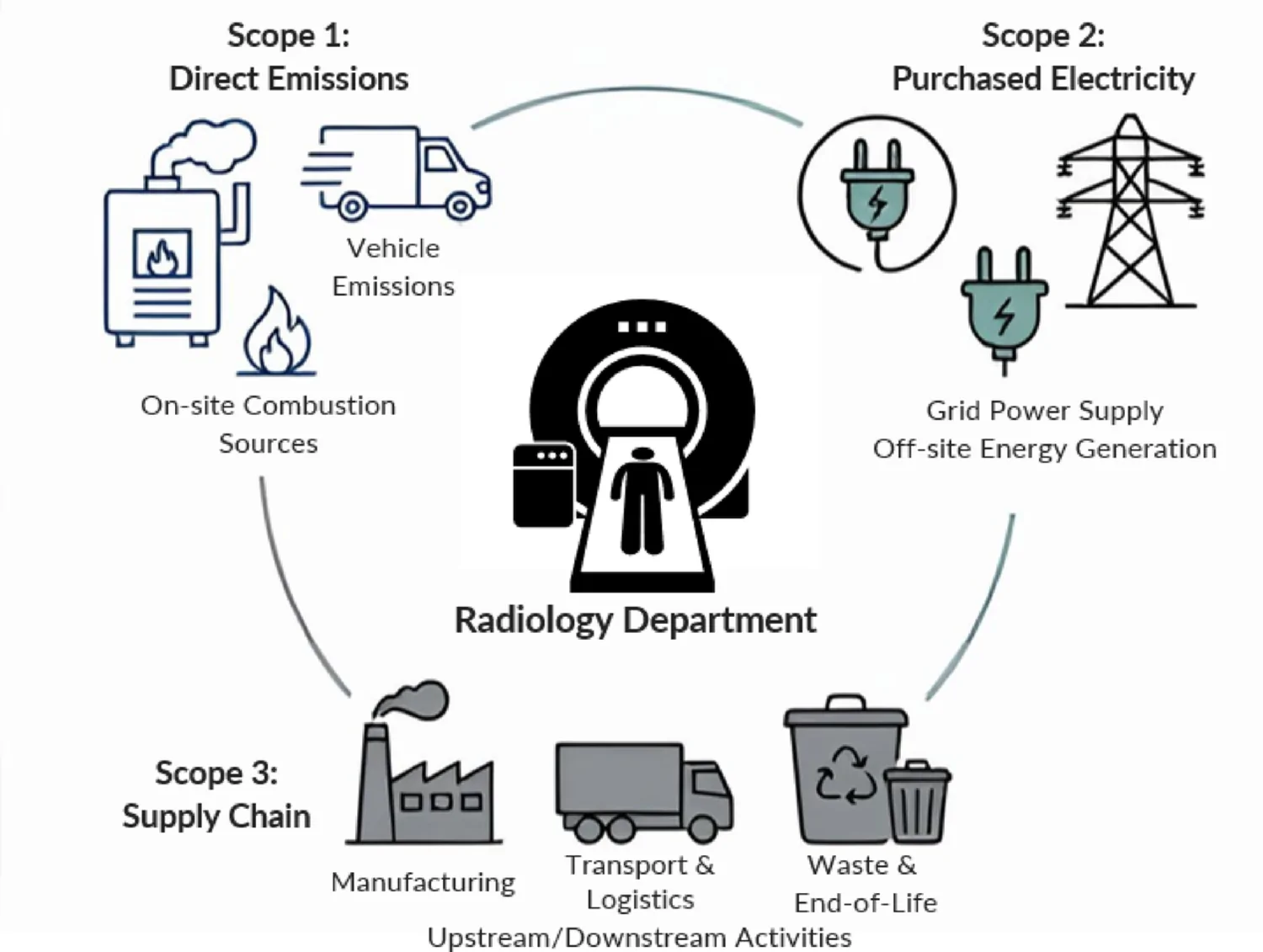
Based on the Greenhouse Gas Protocol, emissions are categorized into three scopes. Scope 1 (direct emissions) includes on-site fuel combustion and facility vehicles. Scope 2 (indirect emissions frompurchased electricity) comprises emissions associated with grid power used for imaging equipment,cooling systems, and IT infrastructure. Scope 3 (other indirect emissions) encompasses upstream and downstream activities across the value chain, including equipment manufacturing, transportation, and waste management. Together, these components define the environmental footprint of radiology services.

To standardize the measurement and reporting of environmental impact, GHG emissions are commonly classified into three scopes according to the Greenhouse Gas Protocol Corporate Standard developed by the World Resources Institute (WRI) and the World Business Council for Sustainable Development (WBCSD) [3, 24] (Fig. 2). Scope 1 refers to direct emissions from sources owned or controlled by an institution, such as facility vehicles, anesthetic gases used in interventional procedures, and on-site fuel combustion. Scope 2 includes indirect emissions associated with the generation of purchased electricity, heating, and cooling used by the institution. Scope 3 encompasses other indirect emissions across the value chain, including equipment manufacturing (embodied carbon), supply chains, pharmaceuticals, waste management, and patient transportation [3].

Applied to the healthcare sector globally, these categories indicate that Scope 1 accounts for 17% of emissions, Scope 2 for 12%, and Scope 3 for the majority at 71% [10]. To our knowledge, explicit scope-specific percentage data for Japan are not available, although a similar pattern appears to be evident in domestic studies. Nansai et al. reported that direct GHG emissions (equivalent to Scope 1) from the Japanese healthcare sector in 2011 were 8.54 megatons of CO2 equivalent (MtCO2e), representing approximately 13.7% of the sector’s total emissions [11]. In contrast, indirect emissions were substantially larger, particularly Scope 2 emissions associated with purchased electricity and Scope 3 emissions related to the procurement of pharmaceuticals and other healthcare products [11]. These findings suggest that, consistent with global trends, reducing power consumption and supply chain–related indirect emissions are likely to be particularly important strategies for mitigating the climate footprint of healthcare in Japan.

**Fig. 3:**
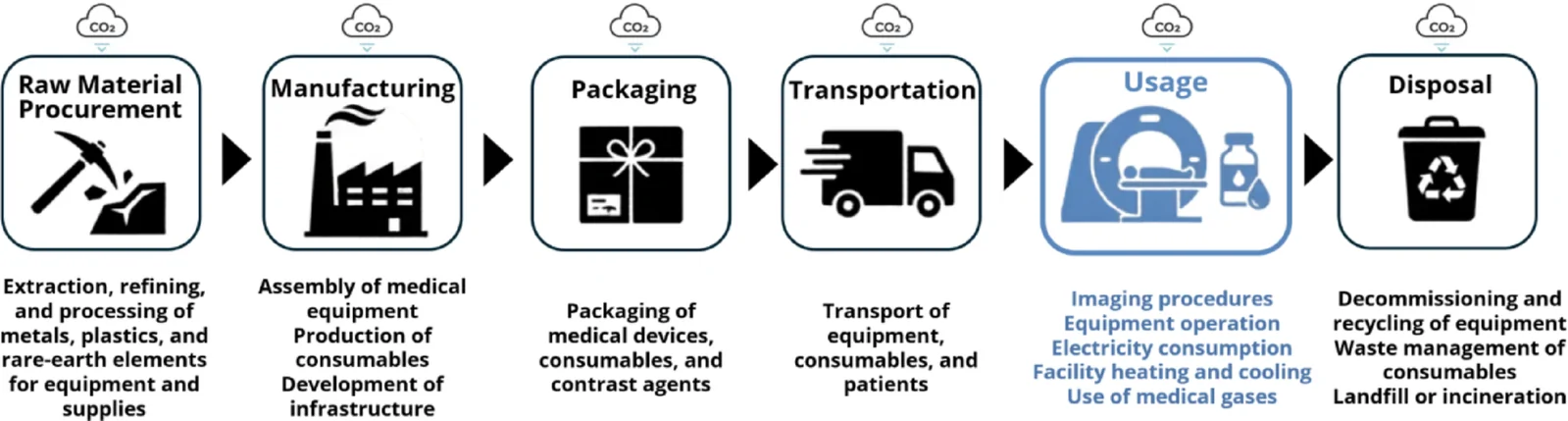
The figure summarizes life cycle stages from raw material procurement to disposal, including key processes during clinical use that contribute to environmental impact

In radiology, LCA has emerged as an important framework for evaluating the environmental impact of imaging equipment and clinical workflows. Based on ISO 14040 standards, LCA assesses the environmental burden of a product or process across its entire life cycle, from raw material extraction and manufacturing to transportation, use, and end-of-life disposal (Fig. 3). In addition to GHG emissions, LCA can evaluate other environmental impact categories, including resource depletion, water use, and ecotoxicity. Recent LCA studies of hospital-based radiology departments have provided quantitative estimates of the environmental footprint of imaging services [25, 26].

A landmark study by Thiel et al. analyzed the environmental impact of diagnostic imaging services at a single large academic medical center and estimated cumulative departmental emissions of approximately 4.6 kilotons of CO₂ equivalent (kt CO₂e) over a modeled 10-year equipment life cycle [25]. Within these estimated emissions, MRI contributed the largest share (approximately 48%), followed by CT (24%), departmental operations including PACS and data storage (12%), and radiography and fluoroscopy (12%) [25].

Similar findings were reported by Carver et al. in an LCA study of three MRI and four CT scanners [26]. Although disposable materials, capital equipment production, and linens contributed to the overall environmental footprint of both MRI and CT services, energy consumption remained the dominant source of emissions, accounting for 58% of the MRI footprint and 33% of the CT footprint [26]. Thiel et al. further showed that modeling a transition to a decarbonized electricity supply using solar power could reduce total departmental emissions by approximately 57% [25].

These findings indicate that, although Scope 3 emissions account for the largest share in the broader healthcare sector, electricity use (classified as a Scope 2 emission) appears to be an important and potentially actionable contributor to the carbon footprint of radiology departments. Consequently, improving energy efficiency and increasing the use of low-carbon electricity sources may represent effective strategies for reducing the environmental impact of medical imaging, highlighting the importance of energy management in radiology departments. In this context, understanding the energy consumption profiles of different imaging modalities is essential.

Although LCA provides a valuable framework for assessing the environmental footprint of radiological services, current evidence has several limitations. Many studies are based on modeling approaches or single-center analyses, which may limit generalizability [25–27]. Furthermore, differences in system boundaries, electricity generation mix, equipment utilization, and life-cycle assumptions can substantially influence the results [9, 25–28]. Therefore, reported values should be interpreted as approximate estimates rather than universally applicable benchmarks.

## Modality-specific energy consumption and the challenge of idle power

**Fig. 4:**
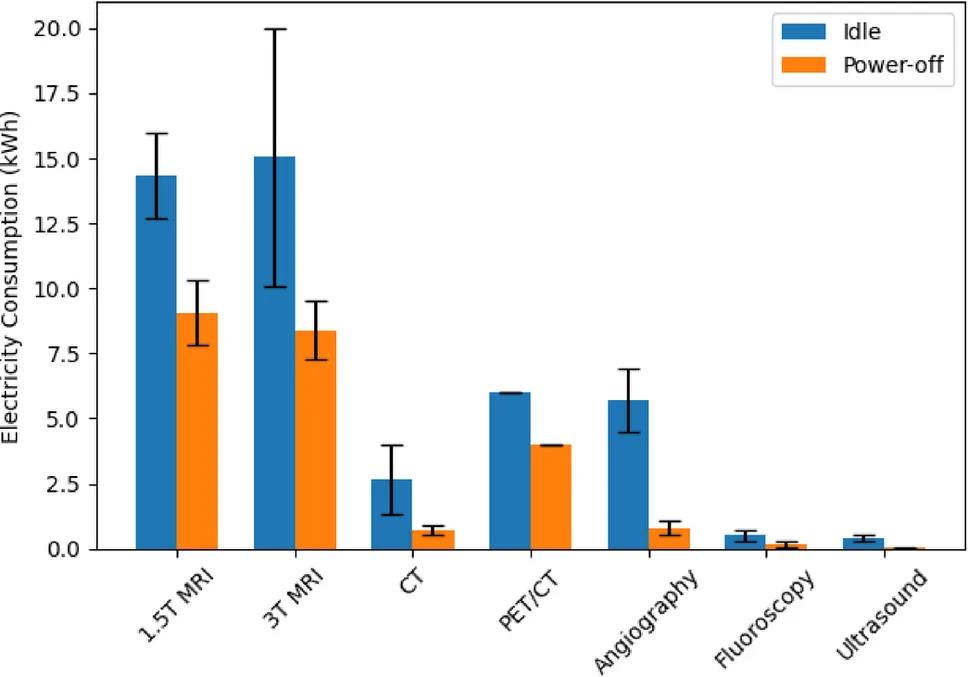
Substantial electricity savings may be achieved by switching imaging equipment from idle to power-off states during non-clinical hours. MRI systems maintain relatively high baseline electricity consumption because of continuous cryogenic cooling requirements; differences between 1.5T and 3T systems likely reflect system-specific design and efficiency rather than magnetic field strength alone. In contrast, modalities such as CT, angiography, and ultrasound demonstrate larger reductions when powered down. Bars represent midpoint values of reported ranges, and error bars indicate the corresponding ranges reported in the literature. Data are derived from previously published studies [ 13, 29, 30, 32, 34]

Energy consumption varies across imaging modalities because of differences in system design, operational requirements, and clinical workflows. Advanced modalities such as MRI and CT require tens to hundreds of megawatt-hours (MWh) annually per scanner for scanner operation, cooling systems, and associated infrastructure [13, 29]. A key characteristic of these systems is the high energy consumption during non-productive (“idle”) periods, when equipment remains powered to maintain system readiness. Idle energy use accounts for a large proportion of total consumption, and switching systems from idle to power-off states can reduce power use by approximately 25% to over 90%, depending on the modality [13, 29, 30]. Differences between idle and power-off consumption across modalities are shown in Fig. 4.

### Magnetic resonance imaging

MRI is one of the most energy-intensive imaging modalities, with energy use strongly dependent on operational state [13, 29, 31]. The highest demand occurs during active scanning and prepared-to-scan modes, while a large amount of energy is also consumed during non-productive periods due to continuous cryogenic cooling requirements. Even in idle or low-power states, MRI systems maintain a continuous and high baseline consumption. Annual electricity use per system ranges from approximately 82.7 to 171.1 MWh, with 72–91% of total energy consumed during non-productive periods [29]. A single MRI examination is also energy-intensive, typically consuming between 12 and 34 kWh of electricity (mean ~ 20 kWh per scan) [13]. Life cycle assessment studies further support this observation, indicating that only about 38% of the total life-cycle energy of an MRI system is used for actual image acquisition and standby operations [32]. These findings highlight the potential for energy savings during idle and off-hours. Replacing older MRI systems with newer energy-efficient models has also been shown to reduce total primary energy consumption by approximately 16.3%, largely due to improved efficiency during non-productive operational modes [33]. However, from a life-cycle perspective, equipment replacement involves important trade-offs. Although newer systems may reduce operational energy consumption, manufacturing, transportation, and end-of-life disposal of imaging equipment are also associated with environmental impacts. Therefore, life-cycle considerations should be incorporated when evaluating the sustainability benefits of equipment replacement. In some situations, upgrading or refurbishing existing hardware and software, rather than complete system replacement, may represent a more sustainable strategy [26, 31]. Careful timing of equipment replacement and upgrades throughout the equipment life cycle may further help maximize environmental benefits while minimizing unnecessary embodied impacts.

Energy consumption is also influenced by technical and operational factors. Although higher magnetic field strengths require greater energy during active scanning because of more powerful gradient and radiofrequency systems [31], differences in non-productive or baseline energy consumption between 1.5T and 3T systems reflect equipment age, system design, and operational practices rather than magnetic field strength alone [26, 34]. Furthermore, newer low-field designs (e.g., 0.55T) may substantially reduce energy and infrastructure demands [31]. In addition, protocol design and sequence selection affect scan duration and energy use per examination [31, 34].

### Computed tomography

CT is another major contributor to energy consumption in radiology. Although its baseline power demand is generally lower than that of MRI, CT remains a significant source of electricity use because of high examination volumes and continuous availability. CT systems exhibit brief periods of high power during scanning, with peak consumption occasionally exceeding 100 kW, but overall energy use is largely driven by non-scanning periods. Approximately two-thirds of daily CT energy consumption occurs when scanners are powered on but not actively scanning [13]. This pattern largely reflects clinical workflow, in which scanners are maintained in a standby “ready-to-scan” state to ensure rapid availability. This is because powering down between examinations has traditionally been impractical due to system warm-up and stabilization requirements.

A typical CT examination requires approximately 1–3 kWh of electricity, depending on the imaging protocol and scanner model [13, 26]; although lower than MRI on a per-examination basis, the high throughput of CT results in cumulative energy consumption reaching approximately 26 MWh annually per scanner [13].

### Interventional radiology (IR)

Interventional radiology (IR) suites and angiography systems are also energy-intensive, largely because they must remain operational around the clock to ensure readiness for emergency procedures. Direct measurements have shown that annual electricity consumption ranges from 3,646 to 26,576 kWh per system, with non-productive periods accounting for 89–99% of total energy use [30].

Beyond imaging equipment, infrastructural requirements further increase environmental impact. LCA studies have reported that up to 49% of total GHG emissions in IR settings may be attributable to heating, ventilation, and air-conditioning (HVAC) systems required to maintain operating-room standards [27]. The production and delivery of single-use sterile supplies represent another major source of GHG emissions. Chua et al. reported that single-use surgical supplies generated 9,640 kg CO₂e over a single week in an academic IR department, accounting for 41% of its total GHG emissions [27]. These findings suggest that strategies such as reducing single-use materials where appropriate, adopting reusable sterile gowns, and improving waste segregation may help mitigate the environmental impact of interventional procedures [8, 27, 35].

## Information technology, PACS, and workstations

The environmental footprint of radiology extends beyond imaging equipment to include digital infrastructure such as Picture Archiving and Communication Systems (PACS), data storage servers, artificial intelligence (AI) processing, and reporting workstations. These systems are essential for daily clinical practice but also contribute substantially to electricity consumption within radiology departments. LCA studies suggest that IT infrastructure accounts for approximately 11–12% of total GHG emissions in a radiology department [25], although the exact proportion may vary depending on institutional infrastructure and workflow.

With the increasing integration of AI and cloud computing in radiology, data processing and storage are often offloaded to external large-scale data centers. These external infrastructures consume large amounts of electricity, with global data centers estimated to account for 1% to 2% of worldwide electricity consumption [21, 36]. From a LCA perspective, the energy costs associated with these external cloud-based systems should be considered when evaluating the environmental footprint of radiology services, particularly as Scope 3 indirect emissions. However, accurately estimating these externalized impacts remains challenging because of a lack of standardized assessment methodologies, the dynamic nature of cloud workloads, and limited transparency from third-party service providers [36]. Developing comprehensive frameworks that capture these external computing costs will be an important area for future research in sustainable radiology.

Reporting workstations are a particularly relevant contributor because of their widespread distribution and prolonged operating times. In the “Green Fingerprint” study, annual electricity consumption across 32 workstations was estimated at 53,170 kWh (roughly equivalent to the annual electricity use of 12 households), with approximately 50% occurring during idle periods [37]. Power management strategies can therefore substantially reduce energy use; implementing automated overnight shutdown policies has been shown to reduce annual consumption by over 30% without affecting workflow [38].

## Strategies for reducing the environmental impact of radiology

Reducing the energy footprint of radiology requires a systematic approach that integrates technological, operational, and clinical strategies (Table 1). Because the present review primarily focuses on energy consumption and GHG emissions, the strategies discussed below mainly target electricity use and idle-period energy consumption while improving workflow efficiency. These interventions may also provide broader environmental, operational, and economic co-benefits through improved resource utilization, waste reduction, and lower healthcare costs.

**Table 1:** Key actions for sustainable radiology and their environmental and clinical co-benefits

Domain	Key actions	Primary target (driver)	Environmental impact	Clinical / operational impact	Implementation difficulty	Cost / resource requirement
Technological	Energy-efficient imaging systems [33, 40] Low-field MRI [31] AI-based image reconstruction [36] Energy-efficient IT infrastructure [25, 38]	Hardware efficiency Computational efficiency	Reduced energy consumption per exam Improved system efficiency [33]	Maintained or improved image quality Shorter scan times [36]	Moderate–High (Equipment and IT dependent)	High (Capital and software investment)
Operational	Power management (shutdown, power-save) [29, 39] Lean-based workflow [42] Reduced idle time [40]	Idle energy consumption Scanner utilization	Reduced non-productive energy use Improved baseline efficiency [29, 40]	Increased throughput Reduced delays and overtime Improved resource utilization [42]	Low–Moderate (Protocol and staff dependent)	Low (Limited direct investment)
Clinical	Reduced low-value imaging [50] CDS and AUC [51] Indication-based protocol optimization [34, 44]	Examination volume Scan duration	Avoided unnecessary imaging Lower per-exam energy and emissions [44, 50]	Improved diagnostic appropriateness Enhanced patient care Reduced downstream testing [51]	Low–Moderate (CDS and governance dependent)	Low–Moderate (CDS and governance resources)
System-level (cross-cutting)	Strengthened governance [22] Regional coordination & resource sharing [7] Integrated sustainability metrics [22]	System structure Equipment distribution Policy environment	System-wide reduction in energy and emissions [7]	Support for SusQI [5] Enhanced coordination and equity [7]	High (Policy and coordination dependent)	Variable (Administrative and planning costs)

Implementation difficulty and cost/resource requirement are qualitative estimates and may vary depending on institutional infrastructure, equipment type, vendor contracts, staffing, electricity mix, workflow, reimbursement structure, and local healthcare system

CDS, clinical decision support; AUC, appropriate use criteria; AI, artificial intelligence; SusQI, sustainable quality improvement

### Equipment power management (technological and operational)

One of the most immediate strategies for reducing energy consumption in radiology departments is powering down equipment when it is not in active clinical use. Heye et al. demonstrated that switching MRI and CT scanners from idle states to “off” or deep power-save modes during nights and weekends can substantially reduce electricity consumption [13, 39]. Given that annual energy costs were estimated at approximately USD 4,700 per CT scanner and USD 24,000 per MRI scanner, such measures may also generate meaningful operational cost savings [13]. Using activity-monitoring dashboards to track equipment status, departments identified systems left powered on unnecessarily and reduced overnight operation, resulting in measurable energy and cost savings [39].

During the workday, software-based approaches can further reduce idle energy consumption. For example, rapid-reactivation power-save modes allow CT scanners to enter low-energy standby states between patients, reducing non-productive energy consumption by approximately 15% without affecting clinical workflow [40].

These approaches demonstrate that relatively simple operational measures can reduce the energy consumption of radiology equipment. However, powering down imaging equipment also has practical limitations. Complete system shutdowns may be operationally infeasible in departments requiring continuous emergency readiness [40]. Furthermore, reactivation from a powered-down state may require several minutes or longer for system warm-up and stabilization [30, 31], potentially affecting workflow efficiency if not appropriately planned. For MRI systems, a baseline level of energy consumption remains necessary because cryogenic cooling systems must continue operating to maintain superconducting magnet integrity and minimize helium loss [34]. In addition, concerns have been raised regarding the potential effects of frequent power cycling on maintenance requirements, equipment reliability, warranty conditions, and system longevity. Although a recent study suggests that structured power-management protocols do not necessarily increase repair rates [30], implementation should be coordinated with equipment manufacturers, maintenance teams, and local operational requirements. Furthermore, some major manufacturers provide guidance on the use of power-save modes during extended idle periods, suggesting that such strategies can be implemented without compromising routine equipment management when appropriately planned [30, 41].

### Workflow optimization and lean management (operational)

Improving workflow efficiency represents an important operational strategy for reducing unnecessary resource consumption in radiology. Lean management, originally developed in the Toyota Production System in automobile manufacturing, focuses on eliminating non–value-adding processes and improving efficiency [42]. In radiology departments, Lean-based workflow optimization may reduce scanner idle time, improve patient flow, and minimize delays between examinations, thereby helping imaging systems to be shut down on schedule at the end of the day. Because CT and MRI systems consume electricity during nonproductive periods, reducing idle time through workflow optimization may also lower energy consumption. A recent study provides a practical example of Lean-based workflow optimization in CT services [43]. RIS action log-guided continuous quality improvement (KAIZEN) interventions were associated with reductions in daily median examination time from 351 to 328 s and patient entry/exit time from 216 to 198 s. The final weekday CT examination also ended 12.49 min earlier, while CT examination volume remained comparable between the baseline and post-intervention periods. Although energy consumption was not directly measured, such workflow optimization may help reduce operational idle periods and facilitate scheduled power-down.

In this way, Lean-based workflow management can simultaneously improve efficiency and reduce environmental impact, consistent with the broader concept of “Lean and Green.”

### Protocol optimization (clinical)

Reducing scan duration can directly decrease energy consumption per examination while also improving patient comfort and increasing scanner throughput. Abbreviated imaging protocols represent one practical approach. For example, studies have shown that replacing comprehensive cardiac MRI protocols with targeted abbreviated protocols can reduce active energy use by approximately 19% while also lowering electricity-related costs through improved scanner utilization and reduced idle time [44]. Another strategy is to modify sequence parameters. Chodorowski et al. developed the “SlowWeAre” neurovascular MRI protocol by modifying parameters such as specific absorption rate (SAR), acoustic noise reduction, gradient settings, and slice thickness [34]. This approach reduced energy consumption by approximately 10–12% [34]. However, such modifications may increase acquisition time and reduce examination throughput, highlighting the need to balance energy savings with operational efficiency.

### Digital innovations and Artificial Intelligence (technological)

Digital technologies, including AI and machine learning, may improve workflow efficiency and reduce scanning time in radiology. AI-based image reconstruction techniques can shorten MRI acquisition times, while AI-driven optimization of CT parameters may reduce both radiation exposure and energy consumption [31, 36]. In addition, AI-based workflow optimization may improve operational efficiency by supporting protocol selection and scheduling, thereby reducing scanner idle time [45].

Large language models (LLMs) have also been explored as tools for supporting sustainability decision-making. Palm et al. evaluated several LLMs—including Claude, ChatGPT, Gemini, and Llama—using standardized questions related to energy efficiency, waste management, and operational policies [46]. The models generally provided practical recommendations, suggesting a potential role in assisting radiologists and administrators in implementing sustainability strategies [46].

However, the rapid proliferation of AI introduces a “sustainability paradox” [36]. Although AI may improve operational efficiency, the computational infrastructure required for model training, deployment, inference, and cloud-based processing can itself generate environmental burdens. Continuous real-time inference may, in some settings, exceed the initial training-related energy use over the life cycle of a deployed model [36, 47]. Consequently, the net environmental impact of AI-assisted imaging remains uncertain at present [47]. It therefore cannot be assumed that introducing AI into imaging workflows will automatically reduce overall energy consumption.

To address these challenges, “Green AI” principles should be translated into practical implementation strategies. Recent literature describes two complementary approaches: green-by AI, in which AI is used to enhance system sustainability, such as reducing scanner energy use through accelerated imaging, and green-in AI, which aims to reduce the environmental footprint of AI models themselves [48]. Practical green-in AI strategies include model pruning and quantization to reduce computational load, transfer learning to avoid training models from scratch, and, where appropriate, federated learning or other deployment strategies that reduce unnecessary data transfer and computation [36, 48]. At the operational level, institutions may consider energy-aware scheduling of non-urgent batch processing, use of low-carbon computing infrastructure where available, and careful selection between cloud-based and local edge-computing deployment according to workload, data-transfer requirements, and local infrastructure [36, 48]. Ultimately, the environmental impact of AI-assisted radiology should be evaluated by balancing potential clinical and operational efficiency gains against the energy and resource demands of model training, deployment, inference, and associated cloud-based computation. Recent literature cautions against the unnecessary use of resource-intensive LLMs for tasks that can be adequately addressed by simpler, more energy-efficient algorithms [49].

### Reducing low-value imaging (clinical)

Reducing unnecessary imaging represents an important strategy for improving the environmental sustainability of radiology. Low-value imaging refers to examinations that provide limited clinical benefit and are not supported by current evidence-based guidelines [2, 50]. Such examinations may also expose patients to unnecessary radiation or trigger additional downstream testing [50].

Previous studies suggest that up to 20% of imaging examinations may be clinically inappropriate [31]. In the US Medicare population, low-value imaging has been estimated to generate excess CO₂ emissions, ranging from 3.55 to 129.2 kilotons annually—equivalent to the electricity-related emissions of a town of approximately 2,000 to 72,000 residents. The wide range reflects uncertainty in the prevalence of low-value imaging and whether standby energy consumption is included in per-examination carbon footprint estimates [50].

Interventions such as clinical decision support (CDS) systems, adherence to appropriate use criteria (AUC), and initiatives such as Choosing Wisely can reduce unnecessary imaging [2, 21, 31, 46]. These approaches have been shown to improve imaging appropriateness and ordering patterns in clinical practice [50–52].

However, addressing overutilization requires recognizing that imaging demand is influenced by complex factors beyond clinical appropriateness alone. Recent literature highlights several drivers of low-value imaging, including medicolegal concerns, defensive medicine, patient expectations, institutional policies, reimbursement structures, local access to imaging equipment, and broader healthcare system characteristics, such as fee-for-service models [47, 53]. These systemic complexities should be considered when designing interventions to reduce unnecessary imaging. Therefore, reducing low-value imaging requires organizational and health system–level approaches in addition to evidence-based guidelines and individual physician decision-making.

When implemented appropriately, reducing low-value imaging may improve patient care while also decreasing energy consumption, medical waste generation, and emissions associated with patient transportation.

## Structural challenges and future perspectives for sustainable radiology in Japan

Beyond operational and technological strategies, structural factors at the healthcare system level, including regional infrastructure, national policies, and local operational practices, also influence the environmental sustainability of radiology.

Japan demonstrates several strengths in sustainable radiology. An Asian Oceanian Society of Radiology (AOSR) survey of 82 Japanese training institutions reported widespread adoption of energy-saving measures, including LED lighting, shutdown of idle PACS workstations, and automated CT shutdown functions [54]. In addition, digital transformation is well advanced, with most institutions operating largely paperless workflows, reducing paper consumption and waste [54].

**Fig. 5:**
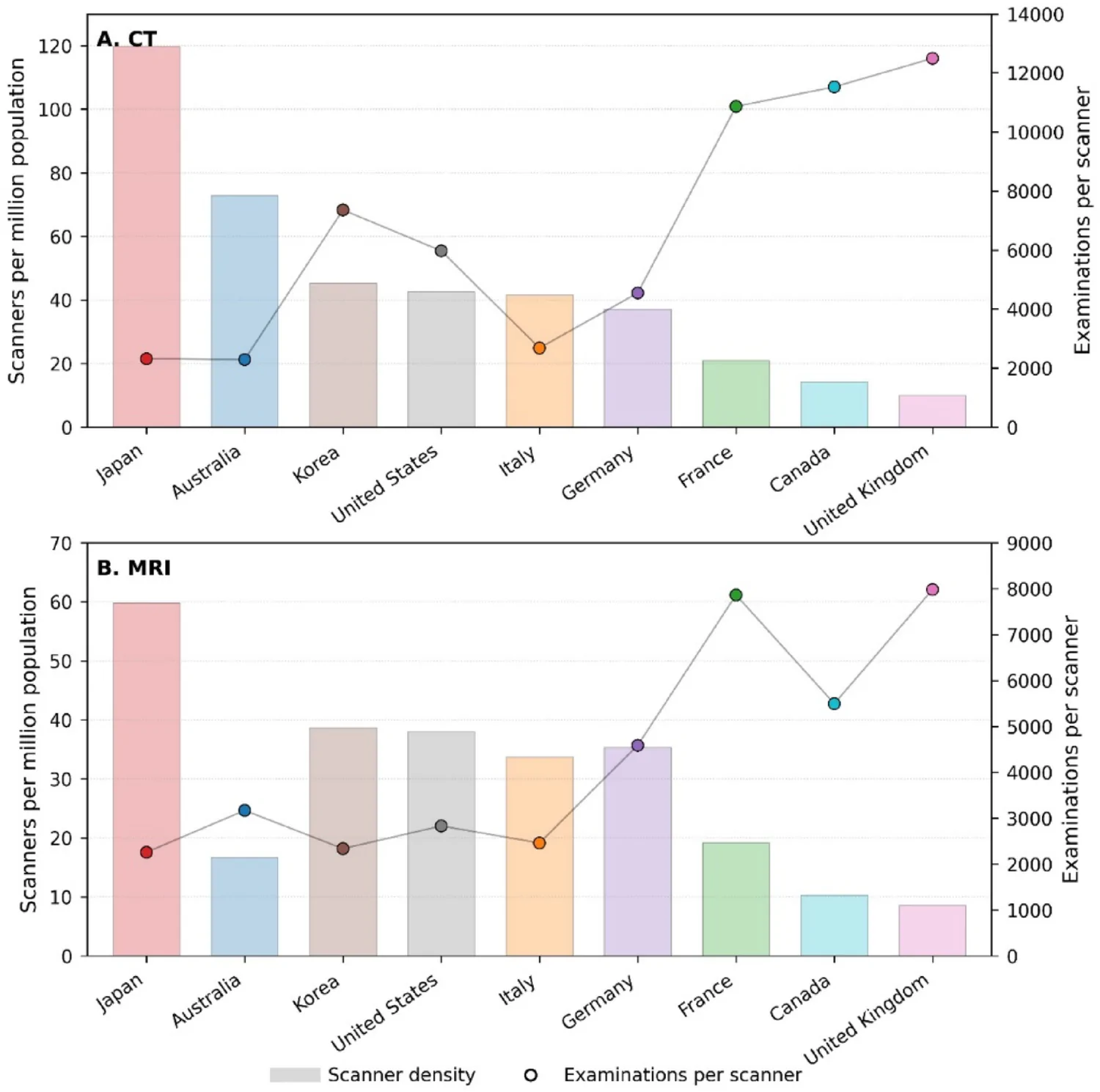
Scanner density and examinations per scanner across selected countries CT and MRI Scanner Density versus Annual Examinations per Scanner across Secondary Medical Service Areas, 2023. **A** CT; **B** MRI. Bars indicate scanner density per million population, and circles indicate annual examinations per scanner. Japan shows the highest scanner density among major developed countries for both CT and MRI, while examinations per scanner are among the lowest, indicating relatively low utilization per unit. Data were obtained from the OECD; data for the United States are from 2021, Canada from 2022, and all other countries from 2023 [55, 56],

**Fig. 6:**
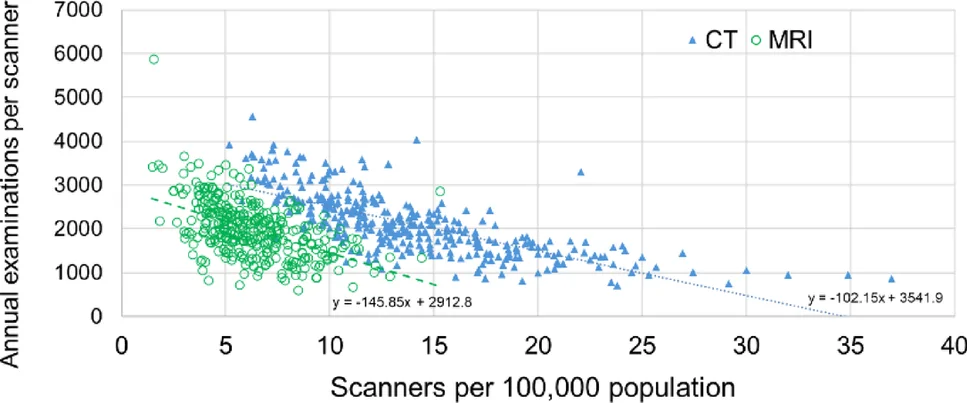
Scatter plots show CT (blue triangles) and MRI (green circles). The x-axis represents the number of scanners per 100,000 population, and the y-axis indicates annual examination sper scanner. Each point corresponds to a secondary medical service area in Japan (2023). Linear regressionlines are shown for each modality. A negative association between scanner density and examinations perscanner is observed for both CT and MRI, indicating that regions with higher scanner density tend to have lower per-scanner utilization. Data were derived from the Ministry of Health, Labour and Welfare, based onthe National Database of Health Insurance Claims and Specific Health Checkups of Japan [56]

However, Japan also faces structural challenges related to the distribution of imaging equipment. Japan has the highest density of advanced imaging equipment among OECD countries, with 111.8 CT scanners and 59.8 MRI scanners per million population in 2023 [55, 56]. Although imaging volumes are also high, examinations per scanner are relatively low compared with other developed countries (Fig. 5), reflecting the wide distribution of imaging equipment across numerous facilities. This pattern is further supported by national data from the Ministry of Health, Labour and Welfare (MHLW), based on 2023 data from the National Database of Health Insurance Claims and Specific Health Checkups of Japan (NDB) [56]. Analyses across regional healthcare service areas (secondary medical areas) show that higher scanner density is associated with lower examinations per scanner (Fig. 6). From a sustainability perspective, this structural imbalance may contribute to increased non-productive energy consumption in Japanese radiology practice.

Importantly, the broad distribution of imaging equipment in Japan should not be viewed only as an inefficiency. It also supports healthcare accessibility, regional equity, and emergency preparedness, particularly in rural and remote areas. Local access to imaging services may reduce patient travel in some settings, whereas excessive fragmentation may reduce operational efficiency. Therefore, the environmental implications of imaging service distribution should be evaluated together with access to care, emergency readiness, and transportation burden [3, 31].

This distributed configuration also poses challenges in the context of limited radiologist resources in Japan [57]. The potential workload for a Japanese radiologist—exceeding 8,000 CT and MRI examinations annually—is approximately three to four times higher than in other developed nations [58]. Physical fragmentation of imaging facilities may lead to less efficient allocation of radiologist expertise across the healthcare system. Although teleradiology supports remote image interpretation, it cannot fully replace locally integrated radiologist involvement in contrast safety, protocol optimization, emergency consultation, quality assurance, and imaging appropriateness.

Governance of imaging appropriateness also remains a challenge. While initiatives such as Choosing Wisely Japan have increased awareness [59], governance varies across institutions. Nationwide data have demonstrated inter-hospital variation in imaging utilization, partly attributable to institutional practice rather than patient-related factors [60]. In addition, a nationwide survey of accredited training hospitals showed that radiologist intervention in inappropriate imaging requests is inconsistent, often limited by time constraints, high reading workloads, and the difficulty of advising referring physicians [57]. Taken together, high imaging volumes, limited radiologist resources, and fragmented equipment distribution may collectively hinder consistent quality control and governance of appropriate imaging.

Addressing these structural challenges will require coordinated approaches that balance sustainability, access to care, and quality of imaging practice. Regional coordination of imaging services, shared resource utilization, and selective consolidation of services, where appropriate, should be considered not simply to reduce scanner numbers, but to optimize equipment utilization and radiologist workforce allocation while maintaining timely and equitable access. When regional imaging hubs are considered, their location and accessibility by public transportation should be included among system-level planning considerations to help reduce transportation-related emissions [3].

Because these approaches are closely linked to healthcare policy and regional planning, long-term, multi-stakeholder collaboration involving healthcare providers, administrators, and policymakers will be essential. Radiologists are uniquely positioned to contribute to these efforts through their central role in imaging utilization and quality assurance.

Strengthening national and organizational frameworks may also help reduce unnecessary imaging and its environmental burden. For example, the Japan College of Radiology (JCR) has recently introduced a self-assessment program to evaluate institutional structures and operational performance in radiology practice. In the future, such frameworks could incorporate sustainability-related indicators, including energy use, scanner utilization, environmental impact metrics, and measures of imaging appropriateness aimed at reducing low-value imaging. Additionally, regional and institutional collaborations are beginning to promote sustainable radiology practices. For example, Kyoto University Hospital and Kyoto Prefectural University of Medicine have jointly launched the Kyoto Radiology Protocol, which outlines seven practical actions for Green Radiology, including appropriate imaging, protocol optimization, equipment power management, resource conservation, waste reduction, education, and cross-departmental dissemination [61, 62]. We highly anticipate—and indeed consider it imperative—that such practical protocols be widely adopted across other institutions, to drive a nationwide shift toward sustainable radiology.

## Conclusion

Radiology plays an essential role in modern healthcare but is also associated with environmental impacts due to the energy demands of imaging equipment, digital infrastructure, and clinical workflows. Life cycle assessment studies indicate that electricity consumption during the operational phase of imaging systems appears to be an important contributor to the carbon footprint of radiology services, with non-productive (“idle”) periods representing a particularly important target for reduction.

Meaningful first steps include monitoring equipment power status, implementing power-save or shutdown protocols during non-clinical hours when feasible, optimizing imaging protocols to reduce unnecessary scan time, improving workflow to reduce idle periods, and strengthening governance to reduce low-value imaging. At departmental and institutional levels, radiology services can incorporate sustainability into routine quality improvement by monitoring equipment energy use, scanner idle time, workstation and IT power management, and imaging appropriateness. At the regional level, scanner utilization and resource allocation should also be considered in planning sustainable imaging services.

Green Radiology will require coordinated technological, organizational, operational, and system-level changes. These efforts should be implemented in a way that maintains diagnostic accuracy, patient safety, and equitable access to medical imaging, while reducing avoidable environmental burden.
